# An open-label, single-arm, phase II trial of buparlisib in patients with melanoma brain metastases not eligible for surgery or radiosurgery—the BUMPER study

**DOI:** 10.1093/noajnl/vdaa140

**Published:** 2020-10-22

**Authors:** Teresa Amaral, Heike Niessner, Tobias Sinnberg, Ioannis Thomas, Andreas Meiwes, Claus Garbe, Marlene Garzarolli, Ricarda Rauschenberg, Thomas Eigentler, Friedegund Meier

**Affiliations:** 1 Center for Dermatooncology, Department of Dermatology, Eberhard Karls University of Tuebingen, Tuebingen, Germany; 2 Health Care Direction, Portuguese Air Force, Lisbon, Portugal; 3 Skin Cancer Center at the University Cancer Centre and National Center for Tumor Diseases Dresden, Department of Dermatology, University Hospital Carl Gustav Carus at the TU Dresden, Germany; 4 Department of Dermatology, University Hospital Carl Gustav Carus at the TU Dresden, Dresden, Germany

**Keywords:** buparlisib, melanoma brain metastases, PI3K inhibitors, targeted therapy

## Abstract

**Background:**

Patients with melanoma brain metastasis (MBM) still carry a dismal prognosis. Preclinical data originated in xenograft models showed that buparlisib therapy was highly effective in therapy-naïve MBM.

**Patients and Methods:**

In this open-label, phase II trial, we investigate the safety and efficacy of monotherapy with buparlisib, a PI3K inhibitor, in patients with asymptomatic MBM who were not candidates for local therapy. These patients had also progressed under immunotherapy if BRAF wild-type or under targeted therapy with BRAF/MEK inhibitors if carrying a BRAFV600E/K mutation. The primary endpoint was the intracranial disease control rate assessed by the investigators. The secondary endpoints were overall response rate, duration of response (DOR) of intracranial disease, overall response, progression-free survival (PFS), overall survival (OS), safety, and tolerability of buparlisib.

**Results:**

A total of 20 patients were screened and 17 patients were treated with buparlisib. Twelve patients had progressed under more than 2 systemic therapy lines and 17 had received at least 1 previous local therapy. There were no intracranial responses. Three patients achieved intracranial stable disease; the median DOR was 117 days. The median PFS was 42 days (95% confidence interval [CI]: 23–61 days) and the median OS was 5.0 months (95% CI: 2.24–7.76 months). No new safety signs were observed.

**Conclusions:**

Buparlisib was well tolerated but no intracranial responses were observed. These results might be explained in part by the inclusion of only heavily pretreated patients. However, preclinical data strongly support the rationale to explore PI3K inhibitor-based combinations in patients with MBM displaying hyperactivation of the PI3K–AKT pathway.

**Key Points**

- Buparlisib is active in xenograft models of therapy-naïve melanoma brain metastases.
- Patients with pretreated melanoma brain metastases did not respond to buparlisib.
- Combination with checkpoint inhibitors in earlier lines could be considered.

**Importance of the Study**

PD-1-based immunotherapy and targeted therapy with BRAF and MEK inhibitors achieve similar initial response rates in intracranial and extracranial melanoma metastases. Still, the majority of patients eventually progress and die of brain metastases, suggesting brain-specific resistance mechanisms. The RAF–MEK–ERK and PI3K–AKT signaling pathways are key players in melanoma progression and drug resistance. Our previous work showed that buparlisib, a PI3K inhibitor, was active in xenograft models of treatment-naïve melanoma brain metastases. Based on this, the BUMPER trial was designed to assess the safety and efficacy of buparlisib in patients with pretreated, progressive melanoma brain metastases, excluded from the ongoing phase III trials. Treatment was well tolerated, with no new safety signs documented, but there were no intracranial responses to buparlisib. Promising new data support the investigation of PI3K inhibitor-based combinations in earlier therapy lines, where the activity of PI3K inhibitors might help overcoming primary resistance to immunotherapy.

Around 80% of stage IV melanoma patients develop brain metastases during the course of their disease.^1^ In recent years, effective treatment options became available for patients diagnosed with advanced melanoma, and at least 3 clinical studies also evaluated these therapies in patients with melanoma brain metastases—the Combi-MB study, the ABC study, and the Checkmate 204 study.^2–4^ Systemic therapy in combination with local therapy, particularly stereotactic radiation and/or surgery, also showed significant benefits in this subgroup of patients.^5–8^ Data from retrospective studies indicate that patients receiving local therapy upfront seem to do better than those who receive local therapy later in the course of the disease.^9,10^ Clinical trials investigating the best therapeutic sequencing are still ongoing.^11,12^

Although systemic therapies achieve similar initial response rates in intracranial and extracranial metastases, the majority of patients eventually progress and die of brain metastases, suggesting brain-specific resistance mechanisms.

The RAF–MEK–ERK and PI3K–AKT signaling pathways are key players in melanoma progression and drug resistance.^13,14^ Preclinical studies have implicated a fundamental role of the PI3K–AKT signaling pathway in both metastatic spread to the brain and survival and growth of melanoma cells in the brain microenvironment. AKT1 has been shown to promote melanoma brain metastasis through regulation and activation of proteins involved in focal adhesion.^15^ Molecular profiling of patient-matched intracranial and extracranial metastases revealed an overall similarity in the majority of molecular features of intracranial and extracranial metastases.^16^ Nevertheless, intracranial metastases demonstrated increased expression of activation-specific proteins in the PI3K–AKT pathway compared to extracranial metastases suggesting a critical role for activation of the PI3K–AKT pathway. Immunohistochemistry of matched intracranial and extracranial metastases from melanoma patients showed identical ERK, p-ERK, and AKT staining patterns. However, hyperactivation of AKT (p-AKT) was seen in brain metastases.^17^ Niessner et al.^17^ also showed that melanoma cells stimulated by astrocyte-conditioned medium showed higher AKT activation than melanoma cells stimulated by fibroblast-conditioned medium. So hyperactivation of the PI3K–AKT pathway in brain metastases is induced by brain-derived factors that may promote survival and drug resistance of melanoma cells in the brain.

Previously, we and others demonstrated that different PI3K inhibitors induced pronounced growth inhibition and apoptosis in melanoma in vitro models.^18^ The PI3K family consists of 4 different classes I–IV. The classifications are based on primary structure, regulation, and in vitro lipid substrate specificity.^19^ Class I PI3Ks have a catalytic subunit known as p110, with 4 types (isoforms) alpha (PIK3CA), beta (PIK3CB), gamma (PIK3CG), and delta (PIK3CD). The full enzymes also contain a second regulatory subunit. Buparlisib is a potent and highly specific oral pan-class I PI3K inhibitor of the catalytic subunits alpha, beta, gamma, and delta, which is able to cross the blood–brain barrier and penetrate into the brain parenchyma.^20^ It shows only reduced potency against VPS34, mTOR, and DNA-PK. Buparlisib inhibited the tumor growth of human BRAF or NRAS-mutant brain metastatic melanoma cells in the brain of mice and also the growth of melanoma cell lines derived from therapy-naïve patients.^18^ Together, these findings suggest that activation of PI3K–AKT signaling is relevant for the survival and therapy resistance of melanoma cells in the brain parenchyma. Using PI3K inhibitor buparlisib could be a strategy to overcome therapy resistance in melanoma brain metastases.

The BUMPER trial is a phase II trial that aimed to assess the efficacy of the PI3K inhibitor buparlisib in patients with melanoma brain metastases not eligible for surgery or radiosurgery.

## Methods

### Patients

The BUMPER study included adult patients (>18 years old) with melanoma brain metastases, for whom a local therapy was not possible or not indicated. Patients with BRAFV600E/K-mutated melanoma needed to have a progressive disease during or after therapy with BRAF/MEK inhibitors. Patients with BRAF wild-type melanoma needed to have a progressive disease during or after receiving immunotherapy. The washout period for previous systemic therapies was 28 days.

Intracranial progressive disease must have been confirmed with magnetic resonance imaging (MRI) prior to the inclusion to the study. Exclusion criteria were, among others, the presence of leptomeningeal involvement and treatment with more than 4 mg dexamethasone or equivalent daily at the time of study entry. More detailed information on the inclusion and exclusion criteria can be found in the study protocol (EudraCT 2013-003306-45; ClinicalTrials.gov Identifier: NCT02452294).

### Study Design and Treatment

In this open-label, single-arm, phase II trial, patients were treated with oral buparlisib 100 mg once daily. A cycle was defined as 28 ± 3 days. Patients were treated until disease progression, unacceptable toxicity, death, withdrawal of consent, or discontinuation from all study treatment due to any other reason.

During the treatment phase, in patients requiring a dose delay of more than 28 days, buparlisib was permanently discontinued. Patients with adverse events (AEs) that required shorter treatment interruptions were allowed to re-start treatment at a lower dose level, once the AE that lead to interruption resolved to Common Terminology Criteria for Adverse Events (CTCAE) grade ≤ 1. Patients requiring more than 3 dose reductions were permanently discontinued from the study. CTCAE grade 4 AEs led to permanent discontinuation, irrespective of recovery time, unless otherwise specified. Patients who permanently discontinued study treatment had weekly follow-ups for 30 days after discontinuation, or resolution of the AE to CTACE grade ≤ 1, whichever occurred first.

### Endpoints

The primary endpoint was intracranial disease control rate (IC-DCR), defined as the proportion of patients with confirmed complete intracranial response (CR), partial intracranial response (PR), or stable intracranial disease (SD) assessed by investigators. The secondary endpoints were overall response rate defined as overall CR and PR, duration of response of intracranial disease, progression-free survival (PFS), overall survival (OS), and safety and tolerability of buparlisib throughout the study.

### Assessments

Assessment of treatment response according to RECIST 1.1 criteria^21^ was done using contrast-enhanced brain MRI and whole-body computed tomography (CT) scans, according to the study protocol specifications. Brain MRI was performed at baseline, and response to therapy was assessed at week 4, week 8, and then every 8 weeks until disease progression or treatment discontinuation. Whole-body CT scan was performed at baseline, and response to therapy was assessed every 12 weeks until disease progression or treatment discontinuation. Different time points of response evaluation for intracranial and extracranial disease were used, as our primary endpoint was IC-DCR in a population with intracranial progressive disease prior to study entry.

Patients with SD, PR, or CR at the time of first evaluation would continue on therapy. Patients with progressive disease would discontinue therapy. Patients who discontinued therapy before disease progression were planned to be followed up until disease progression or death. Safety was evaluated according to the CTCAE version 4.03. Dose reduction, withholding, or discontinuing treatment were based on the discontinuation guidelines presented in the protocol, and the clinical evaluation of the investigator in accordance with the principal investigator.

### Study Oversight

This study was conducted in accordance with Good Clinical Practice guidelines defined by the International Conference on Harmonization and in compliance with the protocol approved by the institutional and ethics board of each participating center; ethics commission of the Eberhard’s Karls University Tuebingen and Technical University Carl Gustav Carus Dresden approval number—719/2014AMG1.

The study was initially conceptualized and developed by the Center for Dermatooncology in Tuebingen and was then expanded to include one additional center at the Technical University Carl Gustav Carus in Dresden. Every patient included provided written informed consent before enrollment. Data were collected and analyzed by the authors.

The first and the corresponding authors wrote the first version of the manuscript. All other authors contributed to the final version published by providing feedback and approved the final version published.

### Statistical Analysis

We assumed a very low disease control rate of 0.10 considering established therapies at the time. Consequently, a rather moderate response rate of buparlisib would justify a further clinical investigation of this drug. Using the 2-stage Simon design for phase II studies, we proceeded as follows: the null hypothesis that the true response rate is 0.125 was tested against a one-sided alternative. In the first stage, 8 patients were accrued. Screening failures were replaced in order to have the minimum number of evaluable patients. An interim analysis for futility was planned after including 8 patients. If there were one or fewer responses, including SD, in these 8 patients, the study would stop. Otherwise, 14 additional patients would be accrued for a total of 22. The null hypothesis would be rejected if 6 or more responses were observed in 22 patients. This design yields a type I error rate of 0.05 and power of 80% when the true response rate is 0.375. Calculations were done with a Simon design calculator presented by the NIH at http://linus.nci.nih.gov/cgi-bin/simonr/cgi_main.

Statistical analyses of the primary endpoint, the IC-DCR, included the entire study population. The survival analyses were performed considering the date of patients’ inclusion on trial. Follow-up time was calculated considering the date of the study’s inclusion and the patient’s last contact or death. Kaplan–Meier estimates were used to conduct the time-to-event analyses. Results are reported as 2-sided *P* values with 95% confidence intervals (95% CIs). The data cutoff date for the analyses was October 22, 2018.

## Results

### Patients and Treatments

The current trial was conducted in 2 centers—the Centers for Dermatooncology in Tuebingen and Dresden. The first patient was enrolled on October 15, 2015 and the current report includes all patients (*n* = 20) who entered the study, all of which had progressed under previous systemic therapies. Seventeen subjects were treated with the study drug and had a radiological evaluation available to assess the primary endpoint. Three patients had a screening failure and therefore did not receive buparlisib. All patients included had multiple melanoma brain metastases (>5), the majority (12/20) had received more than 2 systemic therapies and 17 patients had received at least one previous local therapy. More information on the patients’ characteristics at the time of study entry and an overview of their therapies can be found in Table 1.

**Table 1.** Demographic and Clinical Characteristics of the Patients at Baseline

Baseline Characteristics	Patients Included (*N* = 20)
Sex	*N* (%)
Male	14 (70)
Female	6 (30)
Age at study entry	
Median (min–max)	52 (22–74)
<52 years	8 (40)
52–62 years	9 (45)
>62 years	3 (15)
BRAF status^a^	
BRAF wild type	9 (47)
BRAF mutant	10 (53)
LDH level	
Normal	8 (40)
Elevated	9 (45)
2× >ULN	3 (13)
ECOG PS^a^	
0	12 (71)
1	4 (23)
>1	1 (6)
Presence of neurologic symptoms at study entry	
Yes	0
No	20 (100)
Previous systemic therapies^**b**^	
BRAF inhibitors/MEK inhibitors	17
PD-1-based immunotherapy	24
Ipilimumab	4
Chemotherapy/Other	9
Number of previous systemic therapies	
1	2
2	6
>2	12
Previous local therapies	
STR only	5^c^
Surgery only	0
STR + Surgery	1
WBRT only	6
WBRT + STR	4
WBRT + Surgery	1
No previous local therapy	3

*N*, number of patients in each subgroup; ULN, upper level normal; MBM, melanoma brain metastases; ECOG PS, Eastern Cooperative Oncology Group performance status; PD-1, nivolumab or pembrolizumab; STR, stereotactic radiosurgery; WBRT, whole-brain radiotherapy.

^a^Denotes variables for which the missing/unknown values were excluded from the analysis.

^b^The total number presented here is higher than 20, because all patients have received at least one previous systemic therapy. In some cases, the same patient received more than one therapy line of the same class, that is, targeted therapy, immunotherapy.

^c^Two patients received STR at 2 different time points.

### Efficacy

**Figure 1.**
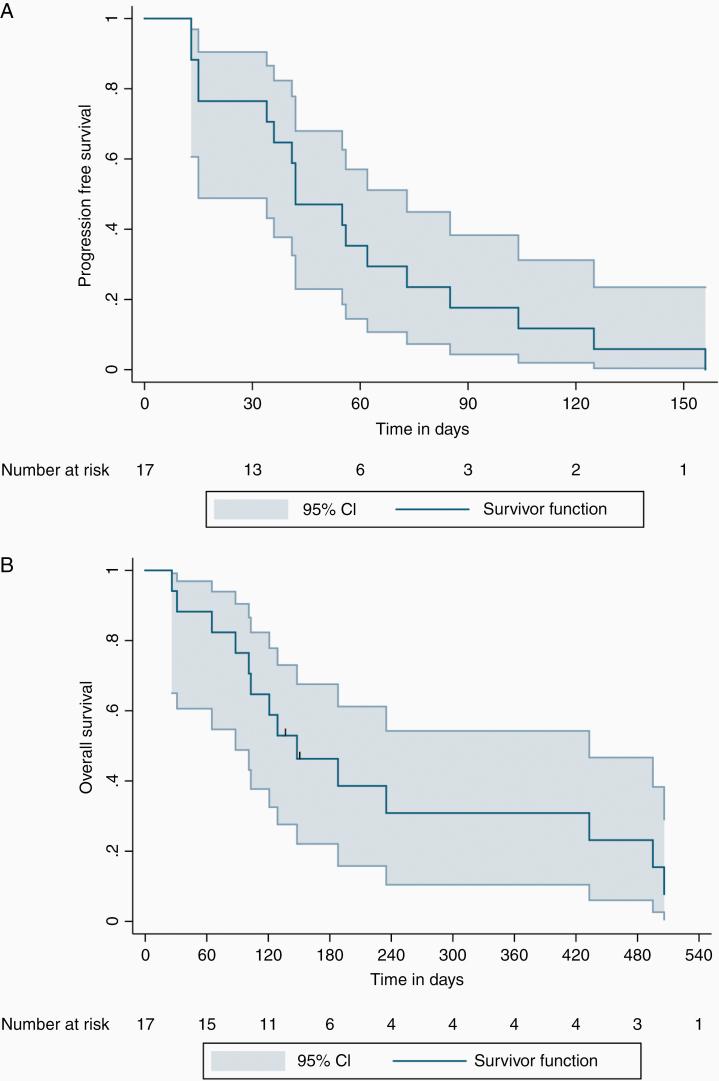
(A) Progression-free survival (PFS) in days for all patients who received therapy with buparlisib. The median PFS in our cohort was 42 days (95% CI: 23–61 days). (B) Overall survival (OS) in months for all patients who received therapy with buparlisib. The median OS was 5.0 months (95% CI: 2.24–7.76 months).

The median time on treatment was 42 days (interquartile range [IQR] = 24–79). The median follow-up time was 135 days (IQR = 94–335). Two patients from the first 8 enrolled had an intracranial SD, implying that the futility criteria at the interim analysis were not fulfilled, and recruitment was continued. Of the 17 patients who had a radiological assessment of response, 3 patients had intracranial SD as the best objective response resulting in an IC-DCR of 17.6%. In those 3 patients, the mean duration of response was 117 days. There were no intracranial CR or PR, and intracranial PD was mostly due to the progression of target lesions. No extracranial responses were observed and all 17 patients had intracranial and extracranial disease progression. The median PFS was 42 days (Figure 1A; 95% CI: 23–61 days). The exact date of death is unknown in 2 cases. The cause of death was progressive disease in all patients included. The median OS was 5.0 months (Figure 1B; 95% CI: 2.24–7.76 months).

### Safety

Those 17 patients who were treated with buparlisib were included in the safety analysis. The investigators reported a total of 62 AEs, with 45 AEs classified as CTACE grade 1 or 2 and 15 AEs classified as serious adverse events (SAEs). Thirteen SAEs were evaluated as unrelated or unlikely related to buparlisib and mainly related to tumor progression. Two SAEs in 2 different patients were considered being related to buparlisib, as follows: (1) erythrodermia and (2) focal epileptic seizure. More information regarding the safety of buparlisib in this trial is given in Supplementary Table 1.

## Discussion

In our population of 17 patients with melanoma brain metastases treated with buparlisib, there was no intracranial response to the therapy. Three patients had an intracranial SD with a duration of response that was less than 4 months. The safety profile of buparlisib in this trial was not different from what was previously reported.^22^ The majority of the AEs were CTCAE grade 1 and 2. There was only one CTCAE grade 4 AE documented in one patient, a focal epileptic seizure that evolved to a generalized seizure, probably related to the underlying brain metastases.

The current study started recruiting patients in 2015 and, at that time, the therapeutic options for patients with melanoma brain metastases were scarce. The phase III trials evaluating systemic therapies in stage IV melanoma excluded patients with melanoma brain metastases.^23–28^ Other trials investigating immunotherapy and targeted therapies specifically in this subgroup of patients were ongoing. However, these trials were recruiting mostly treatment-naïve patients, and the patients included in the BUMPER study would be excluded.^2–4^ This context explains why only heavily pretreated patients were included in this study. The majority (12/20) of the patients had progressed under more than 2 systemic therapy lines. Therefore, our cohort had a worse prognosis compared to the patients included in the mentioned clinical trials. Moreover, 17 patients had received at least one previous local therapy, predominantly whole-brain radiotherapy, which was offered at the time only to patients with multiple brain metastases, that is, more than 3.

Currently, for patients with asymptomatic melanoma brain metastases and therapy-naïve, the recommended first-line therapy is the combination of nivolumab plus ipilimumab.^29^ A recent update of the NIBIT-M2 trial showed that with a median follow-up of 44 months, patients who received combined immunotherapy had a median OS of 29.2 months and a 4-year OS rate of 41%.^30^ Moreover, patients receiving a combination of systemic and local therapies, namely surgery or radiosurgery, seem to have better outcomes than those who do not, regardless of the time point, that is, before or after systemic therapy initiation.^31^ Patients with BRAFV600E/K-mutated melanoma also benefit from treatment with combined BRAF/MEK inhibitors, and if there is a continuous dependency on corticotherapy at the time of systemic therapy initiation, combined BRAF/MEK inhibitors is preferred to immunotherapy.^29^

For patients who progressed upon immunotherapy or targeted therapy, the therapeutic options are still scarce, and further trials with translational research addressing therapy resistance mechanisms are necessary. However, due to the nature of the patients included in this study and the rapid progress of the disease, we had no access to brain metastases samples that could be used for further investigations.

We and others have investigated PI3K inhibitors in patients with melanoma brain metastases.^17,18,32^ Preclinical data showed that the treatment with buparlisib is highly effective in preclinical models of melanoma brain metastases from patient-derived cell lines to xenograft mouse models. Monotherapy with buparlisib reduced proliferation, tumor growth, and induced apoptosis. However, these effects were studied in brain tissue samples that were treatment-naïve. We cannot exclude that local therapy performed, mostly whole-brain radiotherapy, altered the blood–brain barrier permeability, for example, and as a consequence, the intracranial penetration of Buparlisib decreased compared to our preclinical model.

Data on the efficacy of second-line targeted and immunotherapy in stage IV melanoma are limited. However, published works show that the benefit is low, and this is true even for patients who achieve a response and profit from first-line therapy.^33^ Data from our group support this finding, that is, the type of response obtained with the first-line therapy is a prognostic factor, and responses to second-line therapy are modest.^34^ In this study, we only included patients who failed all previous lines of therapy. Therefore, we can assume the presence of resistance mechanisms to previous targeted and immunotherapy.^13,14,35^ Similarly, we cannot exclude the presence of resistance to PI3K inhibitors, which would preclude intracranial but also extracranial response.

Although the effects of the PI3K inhibitor buparlisib did not translate into a clinical benefit in this cohort, several preclinical studies provide a rationale for therapeutic targeting of the PI3K–AKT pathway in melanoma brain metastases. In mouse melanoma brain metastases, a PI3K/mTOR inhibitor modified to optimally penetrate the blood–brain barrier achieved a response in all brain metastases.^36^ In BRAFV600-mutant melanoma brain metastases, a beneficial synergism of PI3K and MAPK inhibition in terms of growth inhibition and cell death induction was seen compared to the monotherapies.^16,32,37–40^ A promising treatment strategy for both BRAFV600-mutant and BRAF wild-type melanoma may be the combination of selective PI3K inhibitors with immune checkpoint inhibitors. Loss of PTEN was associated with a shorter time to brain metastases and a shorter survival in patients with BRAFV600-mutated melanoma.^41^ In melanoma cells, loss of PTEN resulting in increased activation of the PI3K–AKT pathway increased the expression of immunosuppressive cytokines leading to decreased tumor T-cell infiltration and T-cell-mediated tumor cell death.^42^ In mice bearing BRAF-mutant and PTEN-loss melanomas displaying primary resistance to immunotherapy, combining a selective PI3Kβ inhibitor with an agonist antibody of the T-cell costimulatory molecule OX40 significantly delayed tumor growth and extended survival by promoting effector T-cell function and generation of memory T cells.^42^

Altogether, these preclinical data strongly support the rationale to further explore PI3K inhibitor-based combinations in patients with melanoma brain metastases, displaying hyperactivation of the PI3K–AKT pathway.

## Supplementary Material

vdaa140_suppl_Supplementary_Table_S1

## Notes

**Conflict of interest statement**. T.A. reports personal fees and travel grants from BMS, grants, personal fees, and travel grants from Novartis, personal fees from Pierre Fabre, grants from Neracare, and grants from Sanofi, outside the submitted work. H.N. reports research grants from Novartis and Pierre Fabre, outside the submitted work. T.S. reports personal fees from Neracare and research grants from Novartis and Pierre Fabre, outside the submitted work. I.T. reports travel and/or congress registration fees from Abbvie, travel and/or congress registration fees from Novartis, and travel and/or congress registration fees from Roche, outside the submitted work. C.G. reports grants and personal fees from BMS, personal fees from MSD, during the conduct of the study; personal fees from Amgen, grants and personal fees from NeraCare, grants and personal fees from Novartis, personal fees from Philogen, grants and personal fees from Roche, grants and personal fees from Sanofi, outside the submitted work. R.R. received honoraria and travel support from Merck Sharp & Dohme, Novartis, and Amgen, as well as travel support from Bristol-Myers Squibb, outside the submitted work. T.E. reports personal fees from Amgen, grants and personal fees from BMS, personal fees from MSD, grants and personal fees from Novartis, personal fees from Pierre Fabre, grants and personal fees from Roche, grants and personal fees from Sanofi, outside the submitted work. F.M. has received travel support or/and speaker’s fees or/and advisor’s honoraria by Novartis, Roche, BMS, MSD, and Pierre Fabre and research funding from Novartis and Roche. A.M. and M.G. have nothing to report.

## Notes

**Authorship statement**. Study concept: H.N., T.S., C.G., T.E., and F.M.; data collection: T.A., I.T., A.M., M.G., R.R., T.E., and F.M.; data analysis: T.A., T.E., and F.M.; data interpretation: all authors; writing: all authors; final approval: all authors; agreement to be accountable for all aspects of the work: all authors.
