# Supplementary material for: An open-label, single-arm, phase II trial of buparlisib in patients with melanoma brain metastases not eligible for surgery or radiosurgery—the BUMPER study
Source: Neurooncol Adv. 2020 Oct 22;2(1):vdaa140. doi: 10.1093/noajnl/vdaa140 (PMC7712798; doi:10.1093/noajnl/vdaa140)
Supplement: vdaa140_suppl_Supplementary_Table_S1 [file vdaa140_suppl_supplementary_table_s1.docx]

Supplementary table 1: Overview of the reported adverse events in the BUMPER trial

| **Patient ID** | **Reported term for AE** | **CTCAE grade** | **Relationship to study treatment** | **Outcome** | **Serious** |
| --- | --- | --- | --- | --- | --- |
| **1** | Erythrodermia | 3 | Probable | Recovered / Resolved | Yes |
| **1** | Fatigue | 1 | Possible | Recovered / Resolved With Sequelae | No |
| **1** | Cramps in the feet | 1 | Possible | Unknown | No |
| **1** | Rash | 2 | Probable | Unresolved / Not Recovered | No |
| **2** | Worsening general state of health | 3 | Unrelated | Recovered / Resolved | Yes |
| **3** | Fatigue | 3 | Unrelated | Unresolved / Not Recovered | No |
| **4** | Soft tissue infection | 3 | Unlikely | Recovered / Resolved | Yes |
| **4** | Hemiplegia | 3 | Unrelated | Unresolved / Not Recovered | Yes |
| **5** | Vertigo | 1 | Possible | Unresolved / Not Recovered | No |
| **5** | Nausea | 1 | Possible | Unresolved / Not Recovered | No |
| **5** | Arthralgia | 1 | Possible | Unresolved / Not Recovered | No |
| **5** | Flatulence | 1 | Possible | Unresolved / Not Recovered | No |
| **5** | Loss of weight | 1 | Possible | Unresolved / Not Recovered | No |
| **5** | Exanthema maculopapular | 1 | Possible | Unresolved / Not Recovered | No |
| **5** | Diarrhoea | 1 | Possible | Unresolved / Not Recovered | No |
| **5** | Headache | 1 | Possible | Unresolved / Not Recovered | No |
| **5** | Suspected zoster (skin infection) | 3 | Unlikely | Unresolved / Not Recovered | Yes |
| **5** | Worsening psoriasis | 2 | Unlikely | Unresolved / Not Recovered | No |
| **6** | Increased sensitivity of the oral mucosa | 1 | Possible | Unresolved / Not Recovered | No |
| **6** | Dysgeusia | 1 | Probable | Unresolved / Not Recovered | No |
| **6** | GGT increased | 1 | Possible | Recovered / Resolved | No |
| **6** | Creatinin increased | 2 | Unlikely | Unresolved / Not Recovered | No |
| **6** | Anaemia | 3 | Possible | Unresolved / Not Recovered | No |
| **6** | Diarrhoea | 1 | Probable | Recovered / Resolved | No |
| **7** | Urinary tract infection | 2 | Unlikely | Unknown | No |
| **7** | Malignant melanoma metastases | 3 | Unlikely | Recovered / Resolved | Yes |
| **7** | Malignant melanoma metastases | 3 | Unlikely | Recovered / Resolved | Yes |
| **7** | Seizures | 3 | Unlikely | Recovered / Resolved | Yes |
| **7** | Malignant melanoma metastases | 3 | Unlikely | Recovered / Resolved | Yes |
| **7** | Agitation | 1 | Unlikely | Unresolved / Not Recovered | No |
| **8** | Myalgia (cramps) | 1 | Definite | Unresolved / Not Recovered | No |
| **8** | Rash acneiform | 1 | Possible | Unresolved / Not Recovered | No |
| **8** | Tremor | 1 | Definite | Unresolved / Not Recovered | No |
| **8** | Concentration impairment | 1 | Unrelated | Unresolved / Not Recovered | No |
| **9** | Nausea | 2 | Possible | Unresolved / Not Recovered | No |
| **9** | Vomiting | 2 | Possible | Unresolved / Not Recovered | No |
| **9** | Headache | 1 | Possible | Unresolved / Not Recovered | No |
| **10** | Seizure | 3 | Unlikely | Unresolved / Not Recovered | Yes |
| **11** | Nausea | 2 | Possible | Recovered / Resolved | No |
| **11** | Focal epileptic seizure evolving to generalized seizure | 4 | Possible | Recovered / Resolved | Yes |
| **11** | Ocular herpetic infection | 1 | Unlikely | Unresolved / Not Recovered | No |
| **11** | Epileptic seizure | 2 | Unlikely | Recovered / Resolved | No |
| **12** | Fatigue | 1 | Possible | Unresolved / Not Recovered | No |
| **12** | Headache | 1 | Unlikely | Unresolved / Not Recovered | No |
| **12** | Speech disorder | 1 | Unlikely | Unresolved / Not Recovered | No |
| **13** | Dry mouth | 1 | Possible | Unresolved / Not Recovered | No |
| **15** | Right hemiparesis | 3 | Unlikely | Unresolved / Not Recovered | Yes |
| **16** | Diarrhoea | 1 | Unrelated | Unresolved / Not Recovered | No |
| **16** | Anxiety | 1 | Unlikely | Recovered / Resolved | No |
| **16** | Worsening diabetes | 2 | Unlikely | Unresolved / Not Recovered | No |
| **16** | Fatigue | 2 | Unlikely | Unresolved / Not Recovered | No |
| **16** | Cramps | 3 | Unrelated | Recovered / Resolved | Yes |
| **16** | Worsening of general condition | 3 | Unrelated | Recovered / Resolved | Yes |
| **17** | Fatigue | 2 | Unrelated | Unknown | No |
| **17** | Follicular exanthema | 1 | Definite | Recovered / Resolved | No |
| **17** | Diabetes mellitus | 3 | Unrelated | Unresolved / Not Recovered | Yes |
| **18** | Headache | 1 | Possible | Unresolved / Not Recovered | No |
| **18** | Nausea | 1 | Unlikely | Recovered / Resolved | No |
| **18** | Vomiting | 1 | Unlikely | Recovered / Resolved | No |
| **19** | Nausea | 2 | Possible | Recovering / Resolving | No |
| **19** | Hemoptysis | 1 | Unrelated | Unresolved / Not Recovered | No |
| **19** | Loss of appetite | 1 | Possible | Unresolved / Not Recovered | No |

Legend: AE = adverse events; CTCAE = Common Terminology Criteria for Adverse Events. A total of 62 AE was reported, of which 33 Grade 1 AE, 12 Grade 2, 16 Grade 3 and 1 Grade 4 AE. Ten AE were considered not related to the buparlisib, 21 were classified as unlikely related to buparlisib, 24 were considered as possibly related to buparlisib, 4 were considered probably related to buparlisib and 3 were considered as definitely related to buparlisib.
